# Intracranial Aneurysms Treated with a Novel Coated Low-Profile Flow Diverter (p48 HPC)—A Single-Center Experience and an Illustrative Case Series

**DOI:** 10.3390/brainsci15010042

**Published:** 2025-01-03

**Authors:** Nadja Krug, Jan S. Kirschke, Christian Maegerlein, Kornelia Kreiser, Maria Wostrack, Bernhard Meyer, Carolin Albrecht, Claus Zimmer, Tobias Boeckh-Behrens, Dominik Sepp

**Affiliations:** 1 Department of Neuroradiology, University Hospital Heidelberg, 69120 Heidelberg, Germany; 2 Department of Diagnostic and Interventional Neuroradiology, School of Medicine and Health, Technical University of Munich, 81675 Munich, Germany; 3 Department of Radiology and Neuroradiology, University Hospital Ulm, 89081 Ulm, Germany; 4 Department of Neurosurgery, School of Medicine and Health, Technical University of Munich, 81675 Munich, Germany

**Keywords:** flow diversion, intracranial aneurysms, p48 MW HPC, endovascular treatment

## Abstract

**Background/Objectives:** The p48 MW HPC is a novel low-profile flow diverter covered by a hydrophilic polymer coating with antithrombogenic properties, which may reduce ischemic complications and enable a single antiplatelet therapy after insertion of the stent. In this single-center experience, we describe the efficacy of this device, focusing on the illustration of different therapeutic indications and the outcome in various clinical settings with regard to vessel anatomy, bleeding state, and aneurysm configuration. **Methods:** We retrospectively reviewed our database for all patients being treated with a p48 MW HPC flow diverter between February 2019 and July 2021. The efficacy of the treatment was evaluated according to the O’Kelly–Marotta (OKM) scale in the last digital subtraction angiography (DSA) follow-up. Information on complications and medications were collected from our medical records. In addition, to illustrate different indications and clinical settings, we present six of these cases in closer detail. **Results:** 18 aneurysms in 14 patients were treated with the p48 MW HPC flow diverter and in one case with an additional Derivo device. Periprocedural events occurred in 28.6% of the treated patients, which were all successfully resolved within the same session. Follow-up examination information was available for 67% of patients, of which 75% showed complete occlusion of the aneurysm and 83.3% showed a favorable occlusion result (OKM C-D). Two patients with ruptured aneurysms received a single antiplatelet therapy with ASA without thrombotic complications, at least in the short term. New braid deformation patterns were observed in 16.6% at the follow-up examination, but none with subsequent clinical significance. **Conclusions:** The p48 MW HPC is safe and effective in the treatment of a wide spectrum of differently configurated, ruptured, and unruptured aneurysms. Single antiplatelet therapy might be an option in selected cases.

## 1. Introduction

The introduction of flow diverter stents (FDSs) represents a paradigm shift in the treatment of aneurysms. This has enabled the non-invasive and efficient treatment of a wide range of aneurysms [1,2]. Over the past decade, numerous variations of FDSs have been developed, yet they all adhere to a similar structural design: they are made of braided wires with a metal coverage that is approximately 30–40% higher than in conventional stents [3,4]. However, the use of a conventional FDS usually requires strict dual anti-platelet therapy (DAPT), comprising acetylsalicylic acid (ASA) and P2Y12 inhibitors (clopidogrel, ticagrelor, or prasugrel) to prevent thromboembolic complications. This consequently increases the risk of hemorrhagic complications, particularly when FDSs are used in the treatment of ruptured aneurysms.

The p48 MW HPC is a novel low profile FDS with an additional hydrophilic polymer coating (pHPC) with antithrombogenic properties. The objective of this single-center study is to evaluate the efficacy and safety of the device in treating intracranial aneurysms in a variety of clinical scenarios, aneurysm types, and anatomical conditions.

## 2. Material and Methods

### 2.1. Device Description

The p48 MW HPC (WallabyPhenox, Bochum, Germany) consists of 48 braided interwoven platinum-filled nitinol wires coated with a hydrophilic glycan-based multi-layer polymer surface. As it simulates the glycocalyx, this layer has significant anti-thrombogenic properties, as described in a study by Bhogal et al., which showed a lower peak thrombin concentration compared to the p48 without additional coating [5]. Given these characteristics of the p48 HPC, a single antiplatelet medication is justifiable [6]. As an additional feature, there is a central movable wire (MW) with an atraumatic distal nitinol tip to prevent an impaired device opening and any perforation of distal vessels and perforator branches, as well as to provide additional stabilization for the catheter system. The device, which is compatible with a 0.021″ inner diameter microcatheter, was developed for treating vessels with a diameter between 1.75 and 3 mm.

### 2.2. Patient Characteristics and Endovascular Procedures

A retrospective review of our database was conducted for all patients being treated with a p48 MW HPC between February 2019 and July 2021, including patients with ruptured and unruptured aneurysms of various types and locations. Patients who showed a recurrent or remnant filling of the aneurysm following prior surgical clipping or coiling were also included. The dataset included the size, morphology, and location of the aneurysms, the size and location of the FDS, and the complications that occurred during or after the procedure. The degree of angiographic filling of the aneurysm sac and the contrast stasis, as observed in both initial and follow-up images, were assessed using the O’Kelly–Marotta (OKM) grading scale. On this scale, the volume of contrast filling is graded as follows: A—complete (>95%); B—incomplete (5–95%); C—neck remnant (<5%); or D—no filling (0%). The stasis grade is defined as follows: 1—no stasis; 2—moderate stasis; or 3—significant stasis [7]. This grading is more useful for describing the success of aneurysm treatment after flow diverter treatment than other classifications, such as the Raymond–Roy classification, which only considers contrast filling. To demonstrate typical and possible fields of application of this device in a clinical setting, we selected five illustrative and representative cases (Table 1 and Table 2), divided into two groups of ruptured and non-ruptured aneurysms, with detailed descriptions of the procedures (see below).

The decision to treat the aneurysm with a FDS followed the recommendation of the internal neurovascular conference, which consisted of neurointerventionalists and experienced neurosurgeons, and was made after consultation with the patients or their legal representatives about the disease, the procedure itself, the accompanying risks, complications, and treatment alternatives.

A standard bolus of 5000 IU of unfractionated heparin was administered intravenously to all patients during the procedure and, in ruptured cases, after the flow diverter implantation. Furthermore, all flushing solutions were heparinized with 5000 IU/L. Elective patients received 100 mg ASA and 75 mg Clopidogrel for 10 days prior to the intervention. Postinterventional antiplatelet therapy details are shown in Table 1.

All procedures were performed under general anesthesia using a biplane digital subtraction angiography (DSA) system (Philips Azurion) with a 6F or 8F right femoral artery approach. The cervical segment of the internal cerebral artery (ICA) or the vertebral artery (VA) were accessed using a 6F- or 7F-guiding catheter. The target vessel was navigated with a 0.021″ microcatheter (Trevo Pro 18, Stryker Neurovascular, Fremont, CA, USA).

## 3. Results

### 3.1. Patients and Aneurysms

We treated 18 aneurysms in 14 patients (10 female, 4 male). The mean age of the patients was 52 years (range: 26–71 years). Table 1 summarizes the baseline and treatment data, as well as the outcome. Supplemental information concerning details on aneurysm size and the size of the FDS are summarized in Supplementary Table S1. Of these eighteen aneurysms, nine were non-ruptured, five were ruptured, and four aneurysms presented in a setting of acute subarachnoid hemorrhage (SAH) with multiple aneurysms in one patient. Seven lesions were located in the anterior circulation and eleven in the posterior circulation. There were four saccular, four dysplastic, five dissecting, three blister, and two inflammatory aneurysms. The mean dome height was 2.8 mm (range: 0.7–12 mm), the mean neck width was 2.7 mm (range: 1.3–7.2 mm), and the mean aspect ratio was 1.1 (range: 0.5–4.8).

### 3.2. Endovascular Procedures

In all but one case, which included the additional use of a Derivo (Acandis, Pforzheim, Germany), one or more p48 HPCs (see Table S1) were used to treat the aneurysms. Three patients were initially treated with coiling and/or clipping of the aneurysms, and the decision was made to use the FDS due to residual aneurysm sac filling at follow-up (FU).

### 3.3. Early Angiographic Results and Follow-Up

The last available DSA (range: 3–18 months, median: 6.5 months) was used to determine treatment efficacy. In 67% (*n* = 12/18) of aneurysms, a FU DSA was performed. One patient could not receive a FU because he died due to his additional underlying disease (see Case 5: Inflammatory Aneurysm Section). The other missing FUs did not present themselves on the scheduled follow-up date and could not be contacted. A favorable occlusion result was defined as OKM C-D.

Direct postinterventional DSA of the aneurysms showed an OKM score of A2 in 16.7% (*n* = 3/18), A3 in 27.8% (*n* = 5/18), B2 in 16.7% (*n* = 3/18), B3 in 5.6% (*n* = 1/18), C2 in 5.6% (*n* = 1/18), and D1 in 22.2% (*n* = 4/18) of the aneurysms.

In the FU DSA (*n* = 12), the OKM-score was A2 in 8.3% (*n* = 1/12), B2 in 8.3% (*n* = 1/12), and C2 in 8.3% (*n* = 1/12). In 75% (*n* = 9/12) of the aneurysms that had a FU, there was complete occlusion of the aneurysms (OKM = D1), and 83.3% (*n* = 10/12) had a favorable occlusion result (OKM C-D). In 33.3% (*n* = 4/12) with a long-term FU, there was intimal hyperplasia within the p48 HPC without hemodynamic significance or resulting neurological deficits. Intimal hyperplasia was presumed in the case of a locally reduced stent lumen that was progressive compared to the initial findings and could not be explained otherwise. Additionally, we evaluated braid deformation patterns in the FU according to a recent categorization scheme [8]; braid deformation was detected in a total of 16.6% (*n* = 2/12) during FU. Foreshortening occurred in one flow diverter, and fishmouthing and braid dump deformation occurred in another. There was no case in which a secondary treatment was considered to be necessary after the FU procedure.

### 3.4. Periprocedural Complications

Periprocedural events occurred in 28.6% (*n* = 4/14) of the treated patients, and, respectively, in 22.2% (*n* = 4/18) of the treated aneurysms, all due to technical complications. These were resolved within the same session without further complications. No subsequent clinical or neurological deficits were observed in any of the cases; however, it should be noted that, in one case (see Case 5: Inflammatory Aneurysm Section), a comprehensive clinical evaluation was not feasible due to the presence of a serious underlying disease. Detailed descriptions of the complications are provided below:

During the treatment of a saccular AcomA aneurysm of a 70-year-old female patient, excessive shortening of the FDS with only partial coverage of the aneurysm neck was observed; thus, the device was amended with a longer p48 HPC, which enabled optimal positioning.

In another case, shortening of the device occurred due to undersizing of the p48 HPC. Final occlusion of the aneurysm was achieved by delivering a longer Derivo within the shortened p48 HPC.

A further complication, in which a perforation of the aneurysm occurred, is described in detail in Case 5: Inflammatory Aneurysm Section.

During the treatment of a 44-year-old female patient presenting with an anterior communicating artery (AcomA) aneurysm, vasospasms occurred, which were treated effectively with nimodipine.

### 3.5. Illustrative Cases

#### 3.5.1. Unruptured Aneurysms

##### Case 1: Dysplastic Aneurysms

**Figure 1:**
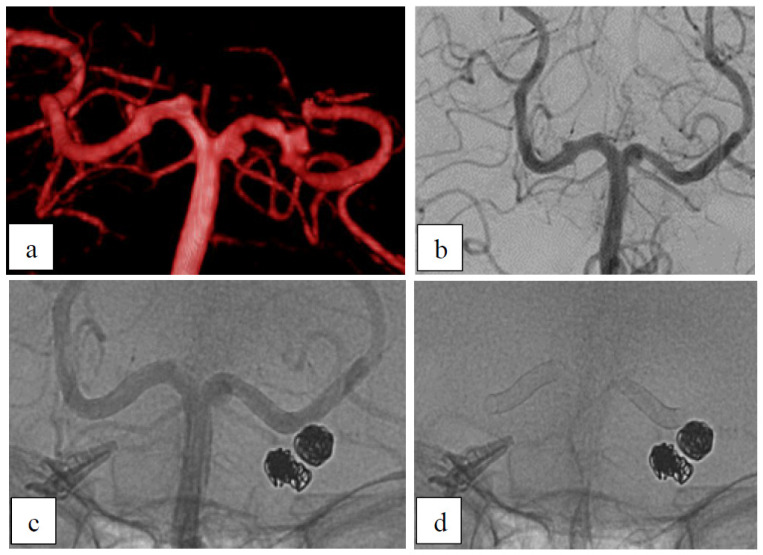
Dysplastic aneurysms. (**a**) Pre-interventional 3D angiogram showing two dysplastic aneurysms of both P1/P2 segments. (**b**) After 3 months, there is no residual inflow into the aneurysm of the left P1/P2 segment. The dysplastic aneurysm of the right P1/P2 segment remained. (**c**) Postinterventional non-subtracted angiogram. (**d**) X-ray showing the implanted p48 HPC device in the P1/P2-segments. There is also a clip and two aneurysms of the anterior circulation packed with coils.

A 50-year-old female patient presented with a diagnosis of multiple aneurysms. After clipping and coiling the aneurysms in the anterior circulation, dysplastic aneurysmatic P1/2-segments of the posterior cerebral artery (PCA) on both sides remained (Figure 1). Each segment was treated in a separate session using a 2 × 15 mm p48 HPC. After the first session (left PCA), the left-sided small aneurysm showed a remnant filling (OKM B3). After 3 months, it was completely occluded and the whole dysplastic left segment reconstructed.

In the second session (right PCA), there was a persistent filling (OKM B2) of the right PCA aneurysm directly post-intervention. A second FU was not available. Despite many important perforators arising from the posterior cerebral artery [9], we did not observe any stent-related stroke.

##### Case 2: Dysplastic Aneurysm with Incorporated Branch

**Figure 2:**
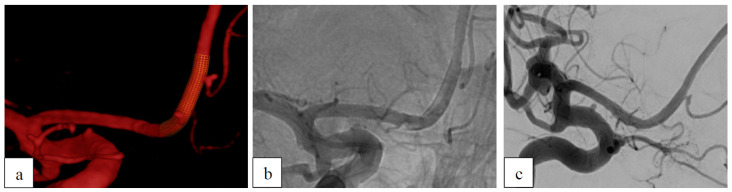
Dysplastic aneurysm with incorporated branch. (**a**) Pre-interventional 3D angiogram showing the two dysplastic aneurysms of the right A2 segment with planned positioning of the FDS. (**b**) Direct postinterventional DSA. (**c**) Follow-up after 6 months shows a completely occluded proximal A2 aneurysm and persisting subtotal filling of the distal aneurysm.

This 55-year-old woman presented with multiple treated aneurysms, one of which was previously ruptured. Among these, there were two non-ruptured, small dysplastic aneurysms located at the right A2 segment of the anterior cerebral artery (ACA) (Figure 2). The frontopolar artery originated from the more distal of the two aneurysms. One p48 HPC 2 × 12 was used for the treatment of both aneurysms. After 6 months, the proximal aneurysm was completely occluded (OKM D1). The distal aneurysm showed a reduced filling (OKM B2).

##### Case 3: Recurrent Aneurysm After Clipping

**Figure 3:**
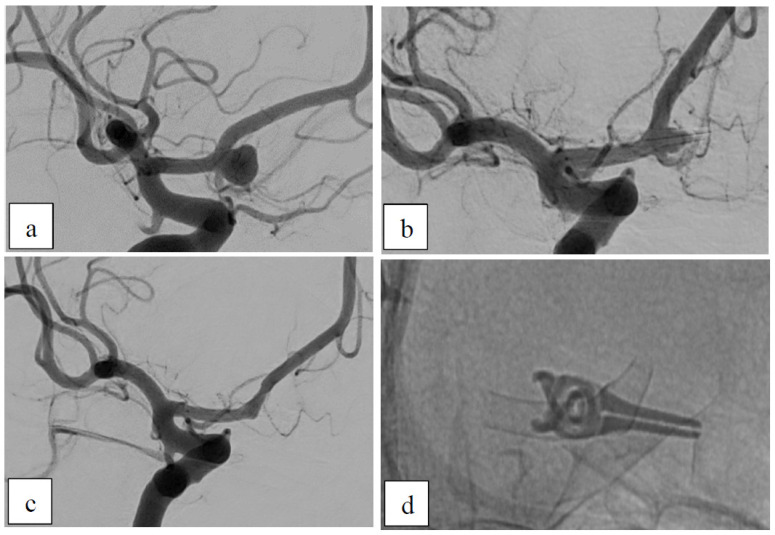
Recurrent aneurysm. (**a**) Right ICA angiogram showing the saccular aneurysm located at the AcomA. (**b**) Pre-interventional angiogram after clipping of the aneurysm and persisting inflow. (**c**) Post-interventional angiogram with complete occlusion of the aneurysm. (**d**) X-ray of the implanted FDS covered by the previously inserted clip.

This 70-year-old female patient presented with a saccular AcomA-aneurysm that was initially clipped (Figure 3). Due to progressive inflow into the aneurysm, additive treatment was recommended. While delivering the p48 HPC 3 × 12 mm, there was excessive shortening of the FDS (parent vessel diameter: 2.1 mm, but the neck of the aneurysm was long and wide), resulting in only partial coverage of the aneurysm neck. A longer p48 HPC 3 × 15 mm was therefore used, providing complete coverage of the aneurysm neck and reduced filling into the aneurysm (OKM B2). After 7 months, the aneurysm appeared completely occluded in the DSA of the right ICA. However, DSA via the left ICA showed a remnant inflow (OKM B2) and minimal intimal hyperplasia in the distal end of the stent, as well as minimal braid dump deformations.

#### 3.5.2. Ruptured Aneurysms

##### Case 4: Dissecting Aneurysm

**Figure 4:**
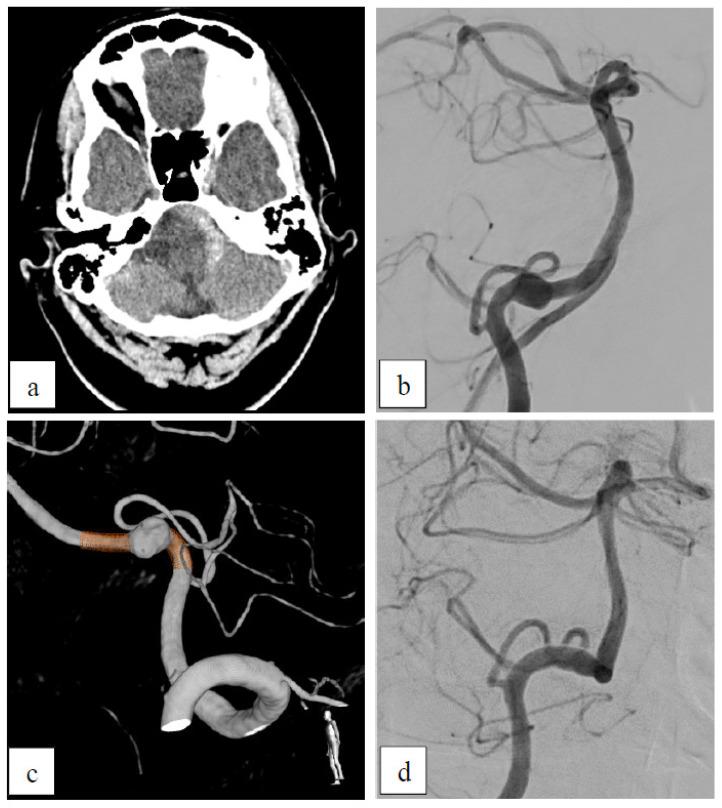
Dissecting aneurysm. (**a**) Native CT scan showing SAH located around the medulla oblongata and the left cerebellar hemisphere. (**b**) Angiogram of the left VA shows the dissecting aneurysm of the V4 segment. (**c**) 3D-angiogram with planned positioning of the FDS device. (**d**) In the FU, after 6 months, there was complete occlusion of the aneurysm.

This 48-year-old patient suffered from a ruptured dissecting aneurysm of the left vertebral artery (VA) (Figure 4). It was treated using a 3 × 18 mm p48 HPC. There was a persisting inflow into the aneurysm sac (OKM A3) in the post-interventional DSA. After all, the 6-month FU DSA showed complete occlusion of the aneurysm (D1).

##### Case 5: Inflammatory Aneurysm

**Figure 5:**
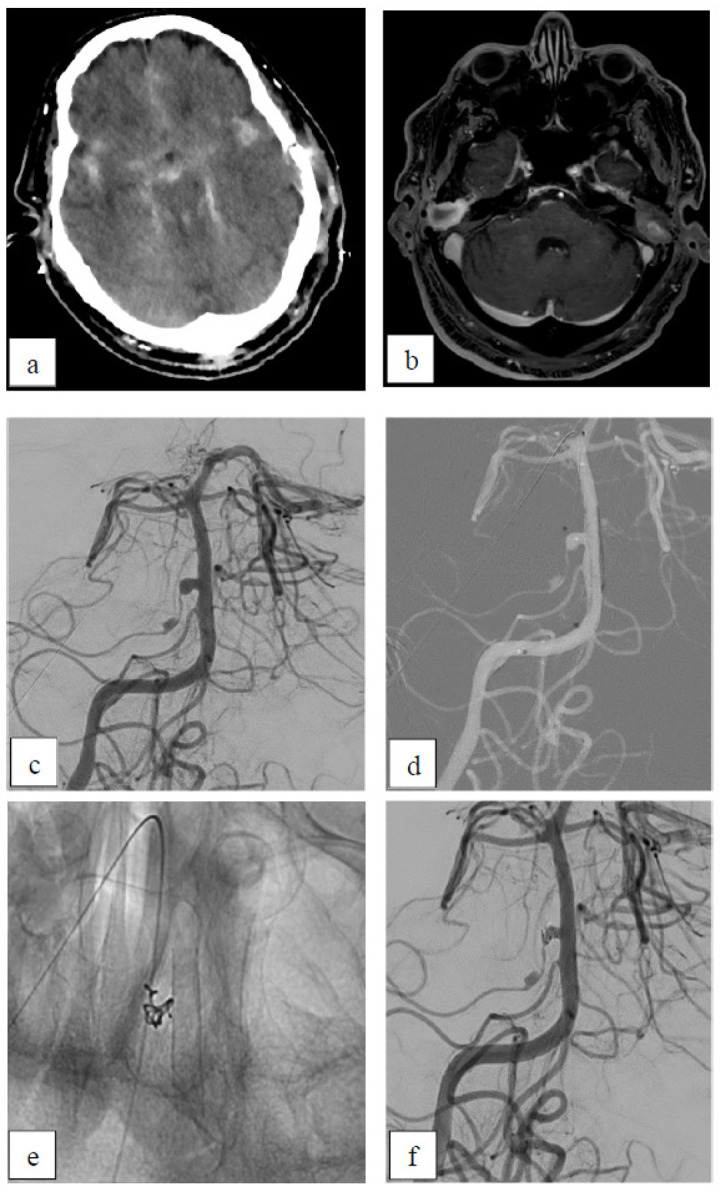
Inflammatory aneurysm. (**a**) Native CT scan showing SAH within the basal cisterns. (**b**) Postcontrast axial T1-weighted MRI showing bilateral mastoiditis. (**c**) Pre-interventional right VA angiogram with the two inflammatory aneurysms of the right AICA. (**d**) Roadmap showing the first delivered p48 HPC and the tip of the microcatheter distally located after perforation of the aneurysm. (**e**) X-ray after coiling of the perforated proximal AICA aneurysm and after implantation of p48 HPC into the BA. (**f**) Directly postinterventional DSA with subtotal filling of the proximal and total filling of the distal inflammatory aneurysm.

This 67-year-old male patient presented with two inflammatory aneurysms (Figure 5) related to a mastoiditis and osteomyelitis of the skull base and concomitant meningitis. The aneurysms were located at the right anterior inferior cerebellar artery (AICA), with one of them adjacent to the basilar artery (BA). When the 3 × 15 mm p48 HPC was delivered through the microcatheter, the second microcatheter within the aneurysm sac (for planned jailing) perforated the proximal AICA aneurysm. Immediately, the FDS was delivered and one hydrocoil was placed within the perforation site and the aneurysm sac, effectively stopping the bleeding. Due to residual filling into the AICA, a second p48 HPC 3 × 9 mm was delivered to cover the aneurysm’s ostium. Directly post-intervention, there was a flow reduction of the AICA aneurysms (OKM B3). An MRI scan was performed 4 days after the intervention. The SAH was comparable to the pre-interventional CT scan as far as possible due to the different modalities, although now, with evidence of blood in the lateral ventricles, this was possibly a result of redistribution. The patient was treated with single anti-platelet therapy until he died 13 days after the procedure due to non-procedural related ventriculitis and meningitis.

## 4. Discussion

In this study, we demonstrated that the novel HPC-coated flow diverter p48 HPC is a safe and effective device for treating aneurysms in a wide range of different aneurysm configurations and locations: 83.3% of the follow-up examinations showed a favorable occlusion result (OKM C-D), and periprocedural complications were resolved without clinical implications.

The most important complications following treatment with FDS are ischemic and/or hemorrhagic complications [10]. According to Texakalidis et al., ischemic episodes occur twice as often as hemorrhagic episodes with a prevalence of 6.6% vs. 3% [11]. The use of coated devices, such as the p48 HPC, may potentially reduce these risks.

In a study by Castro-Afonso et al. that included unruptured aneurysms, ischemic complications were observed in 42.8% (3/7 patients; parent vessel diameter: 2.1–2.7 mm) of cases due to thrombus formation within the p48 HPC after single anti-platelet therapy (SAPT) with ASA [12], which challenges the feasibility of using SAPT in acute cases. In another series, thrombus formation was observed in 50% of cases treated with SAPT following the intervention of ruptured aneurysms. Additionally, thrombosis within the FDS occurred in one of eight patients after three days [13]. It could be concluded from these studies that SAPT is not sufficiently effective to prevent thrombus formation inside the p48 HPC. However, in a separate study, successful prasugrel monotherapy was demonstrated without ischemic complications in 80% of cases [14]. In line with this, our two cases of ruptured aneurysms in which SAPT (with ASA) was used did not present any thrombotic complications, thereby strengthening the notion that this option may be considered in carefully selected cases. Other recent studies have also confirmed the feasibility of SAPT [15,16,17]. One patient presenting with ruptured inflammatory AICA aneurysms was treated with SAPT consisting of 150 mg ASA once a day after being loaded with 500 mg ASA. No thrombotic complications were observed over a period of 13 days, until the death of the patient due to non-procedural related ventriculitis and meningitis. A critical question is which antiplatelet agent to use as SAPT: ASA, clopidogrel, prasugrel, or ticagrelor. In our study, only ASA was used as SAPT, so this question cannot be answered. In a recent meta-analysis from 2023, there was an advantage of prasugrel and ticagrelor over ASA as SAPT found with respect to thromboembolic complications (2.4%/4.2% versus 20.2%) [18]. Overall, the rate of hemorrhagic complications was low. The direct applicability of the above results to the p48 HPC is limited because most of the available studies used different flow diverters [19,20,21]. Therefore, further evaluation in larger studies is needed. In particular, the evaluation of SAPT in the acute setting of SAH remains of great importance.

In our series, 75% of the aneurysms that were followed up were classified as completely occluded at the last follow-up (median: 6.5 months). Other studies have reported a total occlusion of 87% (6-month FU) [22] and of 35% (at the 3- to 15.5-month FU) [23] after treatment with a p48 HPC. In a comprehensive series of 77 aneurysms using the p48 without coating, the latest FU showed complete occlusion in 50% [24]. These relatively moderate midterm occlusion results may be explained by the characteristics of the stent, which is a low-profile FDS with reduced metal coverage, as well as by the antithrombogenic effect of the hydrophilic coating, as has been postulated in other studies [12,24]. Furthermore, the thrombosis inside the aneurysm sac after coverage with a FDS is a gradual process, as demonstrated by the Pipeline for uncoilable or failed aneurysms-trial showing complete aneurysm occlusion rates of 86.8% after 1 year, 93.4% after 3 years, and 95.2% after 5 years [25,26]. It is essential to consider the gradual occlusion process when treating ruptured aneurysms with a FDS and an antiplatelet regimen with DAPT, paying attention to the potential for hemorrhagic complications. In our series, the treated ruptured aneurysms showed lower occlusion rates directly post-intervention compared to the unruptured aneurysms. However, regardless of the administration of DAPT or SAPT, no hemorrhagic complications occurred after the p48 HPC implantation.

Given the gradual occlusion process after flow diverter implantation, the timing of the appropriate follow-up examination must also be reconsidered. Most recommendations and guidelines suggest a FU examination at 3–6 months [27,28]. At that time, a significant proportion of aneurysms will not be fully occluded, as described above. On the other hand, it should be noted that the early detection of emerging braid deformations, such as foreshortening and fish-mouthing, which also occurred in our study over time, is important for preventing late complications. Other modalities such as cone beam CT may also be considered as alternatives or additional options [29].

As the implantation of a flow diverter is technically challenging, there is also a risk of periprocedural complications. This applies also to the p48 HPC, which is mostly used in small vessels. In our study, the peri-interventional events were all due to technical complications, which were resolved in the same session with no new complications. One major complication with perforation of the aneurysm had no clear clinical consequences, although the patient was not sufficiently assessable afterwards due to his severe pre-existing underlying disease. The other patients did not suffer any deficits as a result of the complications. This is in line with other flow diverter studies. A recent study states only 2.5% periprocedural complications occur with p48 HPC [17]. Comparable to our study, the complications could be successfully resolved or had no clinical consequences. Large studies with all types of different flow diverters indicate complication rates of 17%, with a low resulting neurological morbidity of 4.5%, mostly due to ischemia [10].

With regard to the anatomy of the parent vessels, no ischemic complications were observed when this low profile FDS was used in the P1- and P2-segment of the posterior cerebral artery (PCA) with its critical perforators. Illustrative case 2, showing two dysplastic aneurysms of the A2-segment of the anterior cerebral artery (ACA), demonstrated that occlusion of an aneurysm with an incorporated branch can result in persistent inflow into the aneurysm sac. Trivelato et al. suggested that, among other factors, this effect is caused by a pressure gradient which, in the case of branches originating from the aneurysm sac, leads to complete occlusion rates in the 1-year FU in only 60% (vs. 93.1%) [30].

Another important issue is flow diverter braid deformation in the FU and its implications. We had an overall rate of 16.6% braid deformations in the FU, which is in line with a recent study showing a rate of 15% braid deformations [31], but is slightly higher than in a meta-analysis of Ortega-Gutierrez et al. [32]. None of our patients required secondary treatment after FU and there were no associated neurological findings. However, in other studies, retreatments were reported in 17% and permanent neurologic morbidity was observed in 5.5% of patients with braid deformations [31]. This emphasizes the importance of FU examinations, in order to detect braid deformations early enough to prevent neurological consequences.

Our study has several limitations, as follows. First, the number of the included aneurysms is relatively small. Nevertheless, the efficacy of the device was demonstrated in a wide range of different aneurysms including special types of aneurysms. It is not possible to draw any conclusion about the feasibility of SAPT in this context based on only two cases and without long-term FU, even though the observed cases had a favorable outcome with regard to thrombosis.

## 5. Conclusions

The p48 MW HPC is potentially safe and effective in the treatment of ruptured and unruptured aneurysms of different types, which could be demonstrated in a wide spectrum of clinical indications. Single antiplatelet therapy (SAPT) might be an option, at least in selected cases. The outcome of our series is in line with previous reports and adds further evidence to the existing data. However, further research is needed to validate the findings in larger multicenter studies. Our study may be of particular usability for interventionalists due to its illustrative design.

## Figures and Tables

**Table 1:** Baseline, treatment data, and postprocedural outcome.

Age	Sex	Location	Laterality	Type	Ruptured	Previous Treatment	Antiplatelet Therapy	OKM After FDS	Last FU (Months)	OKM Last FU
50	f	P1/2 (two aneurysms)	bilateral	dysplastic	no	no	ASA+ Clopidogrel	left: B2 right: B2	left: 3	left: D1 right: −
55	f	A2 (two aneurysms)	right	dysplastic	no	no	ASA+ Prasugrel	prox: B2 dist: A2	prox: 6 dist: 6	prox: D1 dist: B2
52	m	AcomA	-	saccular	no	coiling	ASA+ Clopidogrel	-	12	D1
26	f	V4	left	dissecting	no	no	ASA+ Clopidogrel	D1	8	D1
71	f	AcomA	-	saccular	no	clipping+ coiling	ASA+ Clopidogrel	C2	6	D1
70	f	AcomA	-	saccular	no	clipping	ASA+ Clopidogrel	D1	7	D1 *
44	f	AcomA	-	blister	yes	no	ASA+ Clopidogrel	D1	13	D1
28	f	P2/P3	right	dissecting	yes	no	ASA+ Clopidogrel	A2	5	D1
71	f	SUCA BA	right	blister saccular	Yes (SAH) †	no	ASA+ Clopidogrel	SUCA:A2 BA: A3	SUCA: 6 BA: 6	SUCA:A2 BA: C2
48	m	V4	left	dissecting	yes	no	ASA+ Clopidogrel	A3	6	D1
60	f	V4	left	dissecting	yes	no	ASA	A3	-	-
67	m	AICA (2 aneurysms)	right	inflammatory	yes (SAH)	no	ASA	prox: B3 dist: A3	-	-
35	f	terminal ICA	right	blister	yes	no	ASA+ Clopidogrel	D1	-	-
57	m	V4	right	dissecting	no	no	ASA+ Clopidogrel	A3	-	-

* There was remnant perfusion into the aneurysm sac via the contralateral left side. † Due to multiple aneurysms, it could not be defined which of the aneurysms was ruptured. Treatment occurred in a setting of SAH.

**Table 2:** Baseline, treatment data, and postprocedural outcome of the cases.

	Age	Sex	Location	Laterality	Type	Ruptured	Previous Treatment	Antiplatelet Therapy	OKM After FDS	Last FU (Months)	OKM Last FU
1	50	f	P1/2 (two aneurysms)	bilateral	dysplastic	no	no	ASA+ Clopidogrel	left: B2 right: B2	left: 3	left: D1 right: −
2	55	f	A2 (two aneurysms)	right	dysplastic	no	no	ASA+ Prasugrel	prox: B2 dist: A2	prox: 6 dist: 6	prox: D1 dist: B2
3	70	f	AcomA	-	saccular	no	clipping	ASA+ Clopidogrel	D1	7	D1 *
4	48	m	V4	left	dissecting	yes	no	ASA+ Clopidogrel	A3	6	D1
5	67	m	AICA (two aneurysms)	right	inflammatory	yes (SAH)	no	ASA	prox:B3 dist: A3	-	-

* There was remnant perfusion into the aneurysm sac via the contralateral left side.

## Data Availability

The data presented in this study are available on request from the corresponding author due to privacy and ethical restrictions.

## Supplementary Materials

The following supporting information can be downloaded at: https://www.mdpi.com/article/10.3390/brainsci15010042/s1: Table S1. Details of aneurysm size and the size of the FDS.
