# Supplementary material for: Intracranial Aneurysms Treated with a Novel Coated Low-Profile Flow Diverter (p48 HPC)—A Single-Center Experience and an Illustrative Case Series
Source: Brain Sci. 2025 Jan 3;15(1):42. doi: 10.3390/brainsci15010042 (PMC11763383; doi:10.3390/brainsci15010042)
Supplement: Supplementary file 1 [file brainsci-15-00042-s001.zip › brainsci-3394521-supplementary.pdf]

Supplementary Materials

Table S1. Details of aneurysm size and the size of the FDS.

Age	Sex	Location	Laterality	Type	Ruptured	Neck Width (mm)	Dome Height (mm)	Parent Vessel Diameter (mm)	Aspect Ratio	FDS
50	f	P1/2<br>(2 aneurysms)	bilateral	dysplastic	no	left: 1.6<br>right: 1.6	left: 1.7<br>right: 1	left: 1<br>right: 1.2	left: 1.1<br>right: 0.6	left: p48HPC 2-15<br>right: p48HPC 2-15
55	f	A2<br>(2 aneurysms)	right	dysplastic	no	prox.: 1.5<br>dist.: 1.6	prox.: 0.7<br>dist.: 0.8	prox.: 2<br>dist.: 2	prox: 0.5<br>dist: 0.5	prox. + dist.:<br>p48 HPC 2-12
52	m	AcomA	-	saccular	no	2	1.7	1.5	0.9	p48 HPC 2-15
26	f	V4	left	dissecting	no	-	-	1.6	-	p48 HPC 3-15
71	f	AcomA	-	saccular	no	2.5	12	1	4.8	p48 HPC 2-12
70	f	AcomA	-	saccular	no	2.7	3	2.1	1.1	initially: p48 HPC 3-12<br>additionally: p48 HPC 3-15
44	f	AcomA	-	blister	yes	1.3	0.9	2	0.7	p48 HPC 3-12
28	f	P2/P3	right	dissecting	yes	-	-	1	-	p48 HPC 2-15
71	f	SUCA<br>BA	right	blister<br>saccular	yes (SAH)	1.5<br>7.2	1.4<br>7	1<br>2	0.9<br>1.5	p48 HPC 3-18
48	m	V4	left	dissecting	yes	6.5	5	2.5	0.7	p48 HPC 3-18 (2x)
60	f	V4	left	dissecting	yes	-	-	2	-	p48 HPC 3-18
67	m	AICA<br>(2 aneurysms)	right	inflammatory	yes (SAH)	prox.: 2.2<br>dist.: 2.5	prox.: 3.3<br>dist.: 1.2	prox.: 2.3<br>dist.: 0.4	prox.: 1.5<br>dist.: 0.5	prox.+dist.:<br>p48 HPC 3-15<br>p48 HPC 3-9
35	f	terminal ICA	right	blister	yes	3	2	3.5	0.7	p48 HPC 3-12<br>Derivo 4-20
57	m	V4	right	dissecting	no	-	-	2	-	p48 HPC 3-18
